# RCT of a client-centred, caseworker-delivered smoking cessation intervention for a socially disadvantaged population

**DOI:** 10.1186/1471-2458-11-70

**Published:** 2011-01-31

**Authors:** Billie Bonevski, Christine Paul, Catherine D'Este, Robert Sanson-Fisher, Robert West, Afaf Girgis, Mohammad Siahpush, Robert Carter

**Affiliations:** 1 Centre for Health Research & Psycho-oncology (CHeRP), Cancer Council NSW & University of Newcastle, Newcastle, Australia; 2 Discipline of Health Behaviour, School of Medicine & Public Health, University of Newcastle, Newcastle, Australia; 3 Centre for Clinical Epidemiology & Biostatistics (CCEB), School of Medicine & Public Health, University of Newcastle, Newcastle, Australia; 4 Cancer Research UK Health Behaviour Research Centre, Department of Epidemiology and Public Health, University College London, London, UK; 5 Department of Health Promotion, Social and Behavioral Health, College of Public Health, University of Nebraska Medical Center, Nebraska Medical Center, Omaha, USA; 6 Deakin Health Economics, Deakin University, Burwood, Victoria, Australia

## Abstract

**Background:**

Disadvantaged groups are an important target for smoking cessation intervention. Smoking rates are markedly higher among severely socially disadvantaged groups such as indigenous people, the homeless, people with a mental illness or drug and alcohol addiction, and the unemployed than in the general population. This proposal aims to evaluate the efficacy of a client-centred, caseworker delivered cessation support intervention at increasing validated self reported smoking cessation rates in a socially disadvantaged population.

**Methods/Design:**

A block randomised controlled trial will be conducted. The setting will be a non-government organisation, Community Care Centre located in New South Wales, Australia which provides emergency relief and counselling services to predominantly government income assistance recipients. Eligible clients identified as smokers during a baseline touch screen computer survey will be recruited and randomised by a trained research assistant located in the waiting area. Allocation to intervention or control groups will be determined by time periods with clients randomised in one-week blocks. Intervention group clients will receive an intensive client-centred smoking cessation intervention offered by the caseworker over two face-to-face and two telephone contacts. There will be two primary outcome measures obtained at one, six, and 12 month follow-up: 1) 24-hour expired air CO validated self-reported smoking cessation and 2) 7-day self-reported smoking cessation. Continuous abstinence will also be measured at six and 12 months follow up.

**Discussion:**

This study will generate new knowledge in an area where the current information regarding the most effective smoking cessation approaches with disadvantaged groups is limited.

**Trial registration number:**

ISRCTN: ISRCTN85202510

## Background

### The high burden imposed by smoking in socially disadvantaged groups

Quitting smoking is the single most important preventive health behaviour significantly reducing risk of morbidity and premature mortality [1]. Disadvantaged groups are an important target for smoking cessation intervention. Social disadvantage may be defined in terms such as low socioeconomic status or poverty, or less easily quantified indicators of deprivation such as not having adequate and affordable housing; or being subject to discrimination [2]. Smoking rates are markedly higher among severely socially disadvantaged groups than in the general population where rates are declining [1]. In Australia, smoking rates have been found to be highest amongst the following groups; Aboriginal and Torres Strait Islander people (50%), the homeless (70%), people with a mental illness (60%), people with drug and alcohol problems (74% to 100%), vulnerable youth (65%), those in prison (90%) and low-income single mothers [3-6]. These data reveal that the poorest and most marginalised members of society are the most likely to be smokers and at highest risk of smoking related disease.

### The personal and environmental context of smoking and quitting in disadvantaged groups

Qualitative research has shown that smoking is often deeply embedded in the lives of disadvantaged smokers because they live, socialise and/or work with other smokers, making it more difficult to refrain from smoking [7]. It has also been shown that smoking is influenced by unfavourable life trajectories such as childhood and educational disadvantages [8]. Smokers from more deprived socioeconomic groups also have higher levels of stress which can play a role in relapse because smokers often use smoking to cope with stressful aspects of their lives [9]. Importantly, studies have described the higher rates of nicotine dependence in smokers from more deprived socioeconomic groups [10], and past research has found nicotine dependence is a predictor of failure of attempts to stop smoking [11]. These factors suggest that smokers from lower socioeconomic groups may need strategies that address their unique life circumstances.

Studies have found that socially disadvantaged smokers are as interested in and as motivated to quit as other smokers [12,13]. However, some important differences in quitting behaviours between socioeconomic groups exist. Lower socioeconomic groups tend to receive less physician advice to quit, [14] access telephone quitlines less, even during mass media campaigns [15] and use fewer pharmacotherapies [16]. Also, although the number of quit attempts is similar across groups, it is achieving quitting success that is difficult for those in lower socioeconomic groups [17]. It appears smoking and smoking cessation in disadvantaged groups are complex behaviours with deeply embedded interacting influences. Multifaceted and intensive interventions addressing multiple motivators may be appropriate approaches for further research.

### Evidence regarding the effectiveness of smoking cessation interventions targeting the socially disadvantaged

The efficacy and effectiveness of a range of cessation interventions have been repeatedly and rigorously evaluated for the general population, including brief advice from health professionals, counselling, and pharmacotherapy [18]. However, there is a paucity of methodologically rigorous trials evaluating smoking cessation interventions among the socially disadvantaged. Three reviews [19-21] specifically targeting socially disadvantaged populations were unable to identify smoking cessation interventions with sufficient evidence supporting their efficacy or effectiveness in the groups examined. More recently, Murray et al [22] have reviewed the literature describing strategies to improve access to smoking cessation services for disadvantaged groups. Tailoring interventions and making them more 'client-centred', providing cessation support at accessible settings and using established cessation strategies improved proxy outcomes such as intention to quit and attitudes towards smoking. Although the overall trend of the results showed promise, the studies displayed several limitations such as small sample sizes, observational designs, use of proxy 'smoking behaviour and intent' outcomes and lack of biochemically verified smoking cessation outcomes [22].

### Community social service setting for reaching the socially disadvantaged

Non-government, community based social and welfare agencies are a suitable setting for reaching vulnerable sub-groups of the population. They access a high proportion of the groups with high smoking rates on a regular basis, are increasingly open to providing cessation support, are a trusted source of support and provide or liaise with a range of auxiliary support services that address additional life issues known to affect relapse [23]. In New South Wales (NSW) Australia, there are over 7,000 community service organisations assisting over 85,000 clients a year [23]. Clients include those most affected by smoking; low income families, the unemployed, Indigenous Australians, those with mental health concerns, marginalised youth and people with addiction problems. Overall, the services estimated that over half (56%) of their clients were current smokers [24]. Local cancer authorities have recognised the potential of this setting to reach disadvantaged smokers [3,25]. One study used a qualitative evaluation to test the potential of a smoking cessation group program in three community social services; a residential drug and alcohol treatment centre, an Aboriginal health service and a mental health service [25]. A key feature of the intervention involved the use of an algorithm allowing for individually tailored combinations of NRT for 'hard to treat' smokers [26]. The study, which was limited by a number of factors found high rates of smoking cessation (32% validated self report at 6 months follow-up) [25].

### Study Aims

This study aims to evaluate the efficacy of a client-centred, caseworker-delivered cessation support intervention at increasing smoking cessation rates in a socially disadvantaged population.

## Methods/Design

### Study Design

A block randomised controlled trial will be conducted. At baseline, clients attending one community social service will be asked to complete a touch screen computer survey asking them about health issues and their smoking status. Clients identified as smokers will be asked to participate in a smoking cessation trial. Allocation to the intervention or control groups will be determined by time periods with clients randomised in one-week blocks to reduce selection bias and contamination between groups. The trial has been carefully designed to conform to the CONSORT statement which has recently been updated for psychosocial and behavioural interventions [27].

The trial is funded by the National Health and Medical Research Council (NHMRC) of Australia (Ref no. 631055) and approved by the University of Newcastle Human Research Ethics Committee (Ref no. H-2010-1002).

### Setting

The study will be conducted in a non-government organisation, Community Care Centre (referred to here as the Centre) which is a community social service located in NSW, Australia providing financial counselling, relationship counselling, Life Skills courses, emergency relief and a communal use room/waiting area. The Centre operates 7 days a week, providing counselling services 4 days a week and predominantly assists those on government income assistance (95% of clients).

### Sample

Clients eligible for the trial will be those aged over 18 years, attending the Centre for their first visit, and who are self-reported smokers. Clients who have attended the Centre at another time in the past and are returning to start a new program will be eligible to participate. English-speaking clients who are unable to read at this level will be offered assistance from the research assistant (RA) to complete study consent forms and surveys. *Ineligible clients *will be those who are non-English speaking, presentation with uncontrolled mental illness (as identified by the RA and confirmed by the caseworker), and whose partner is already enrolled in the study.

### Sample size

A sample of 200 participants per group at 12 months will allow detection of a difference in smoking cessation rates of 8% between groups at 12 months (slightly smaller difference of 7% can be detected at 1 month), based on 5% smoking cessation in the usual care group and 13% in the intervention group, at 5% significance level and 80% power. This effect size is achievable and important from a population-effect perspective. Non-randomised and observational studies using less intensive smoking cessation interventions within disadvantaged groups have demonstrated high cessation rates [25,28-30]. Estimating that 30% of the sample are lost to follow-up (10% at each of one, six and 12 months), 290 patients per group will be recruited at baseline. The Centre receives approximately 50 new clients a week and 50% of those are for ongoing counselling care. All new clients will be eligible to complete the baseline touch screen computer survey. Over a 12-month recruitment period, 1,300 new clients will attend for counselling services. Staff estimate that 60%-90% of clients are current smokers. Assuming a median value of 75%, there will be approximately 975 eligible clients during the study period. Assuming a consent rate of 60% [28-31], 585 individuals will consent to participate in the study.

### Recruitment and randomisation

A trained RA will be situated in the Centre communal waiting room during recruitment. The RA will approach all clients entering the Centre, ascertain eligibility, and seek written consent. A two-stage consent process will be used. First, the RA will inform clients that the study aims to expand the services provided by the Centre and involves completing a health survey on a touch screen computer and that their case-worker may discuss health issues with them. When they complete the survey, if they reported to smoke, they will be informed, on-screen, that a study for smokers is being conducted and that the RA will seek their consent to participate in the smoking study. They will be told that participation in the smoking study will involve their counsellor or case worker possibly discussing quitting with them, and that they will be asked to return to the Centre in one, six and 12 months time to complete the survey and conduct a breath test for carbon monoxide (CO) measurement. Consenting clients will be assisted in the use of the touch screen survey. The RA will review the clients' smoking status on a touch screen survey print-out and randomly allocate participants who are current smokers to the control or intervention group depending on whether it is an intervention or control week. Intervention or control times will be randomly allocated in blocks of four by a computer generated randomisation procedure. Clients who are not smokers will not participate further in the study.

### Touch screen computer baseline survey and print-outs

Studies indicate that touch screen computers are a confidential, acceptable, feasible, user-preferred, and cost-effective mechanism for collecting health information in vulnerable populations [32-37]. The computer will be placed in a private location ensuring confidentiality. The RA will be present at all times when the computer is in use. An additional advantage of the computerised data collection is that it can provide 'real time' results, immediately available to users and staff [33]. The computer will provide intervention participants with two print-outs of their results with a checklist that will outline components of the intervention their case worker should address. One copy will go to the case worker and one will be retained by the client. Clients and staff will be asked to keep checklists and mark off the components of the intervention 1) offered, 2) accepted or refused by the client, and 3) provided. These print-outs will form one component of the intervention as well as process measures.

### Definition of current smoker

The survey will assess demographic characteristics, client smoking status as well as a number of screening tools (such as nicotine dependence, partner's smoking status, depression, financial stress). Self-reported smoking will be determined using the following questions "Which of the following best describes your smoking status?" and respond by selecting from "I'm a smoker, I smoke daily", "I'm a smoker, I smoke occasionally", "I'm an ex-smoker, I never smoke now" or "I'm a non-smoker, I have never smoked." *Clients who smoke daily or occasionally will be asked if they have smoked in the past 7 days and those who have will meet the definition of current smokers for the purpose of this trial. *The use of these items to elicit smoking history has demonstrated accuracy in past research [38].

### Experimental groups

#### The minimal ethical care (control) group

Consenting, smoking clients who complete the survey during control weeks will receive on-screen information at completion of the survey including advice to quit and the telephone smoking cessation assistance Quitline number.

#### The smoking cessation (intervention) group

will receive an intensive client-centred smoking cessation intervention offered by the caseworker over one to two face-to-face and two telephone contacts.

### Intervention

The theoretical framework for the intervention is based on the PRIME theory of motivation [39]. A number of factors are likely to influence an individual's motivation to smoke and quit [7-17,25]. The theory suggests that in order to motivate change, an intervention must minimise the strength and frequency of the need to smoke, maximise the countervailing desire to remain abstinent, optimise use of medication and maximise the capacity to exercise self control. The intervention aims to: use motivational interviewing [40] to encourage repeated quit attempts among all smokers, maximise use of effective quitting assistance strategies [18] and provide support for life 'stresses' believed to contribute to the high rates of relapse in disadvantaged populations [9-11].

Tailoring will be used as cessation research with disadvantaged groups suggests that in order for the client group to benefit, the intervention must fit their level of need, their unique circumstances and be accessible [22,25,39,41]. Data from the baseline screening survey regarding nicotine dependence, previous quit attempts, depression, partner smoking status, and financial stress will be used to produce an individualised checklist of the types of assistance each client may need.

Other behavioural elements of the intervention program will include use of motivational interviewing, behavioural contracting, provision of pharmacotherapy subsidies, allocation of a support person and support pack, referral to specialist quit services as well as Centre-run Life Skills courses, and follow-up using unscheduled drop-in or phone-in sessions.

#### The Case Worker Sessions

The intervention will be implemented over one or two face-to-face visits (each two weeks apart) which will commence immediately following baseline survey completion, followed by at least two phone contacts (one week apart). This intervention will constitute an add-on to clients' usual regular counselling visits, reducing additional costs to the Centre and to clients. If a client requires further contact, staff will provide further quitting assistance and record what they delivered on their checklist. Clients who do not return within the two week timeframe will be contacted by the caseworker by telephone. Appendix 1 provides a session by session description of the intervention.

#### Staff training and resources

Centre staff will be provided with training in the smoking cessation strategies. Local training will be provided during a one day course. Similar courses are regularly provided for various health professionals. The course will be tailored to the needs of community service staff and clients.

### Measures

#### Primary outcome measure - *client validated self-reported smoking cessation*

There will be two primary outcome measures obtained at one, six and 12 month follow-up: 1) 24-hour CO validated self-reported smoking cessation and 2) 7-day self-reported smoking cessation. These follow-up time periods and measures of smoking cessation have been recommended by the Society for Research on Nicotine and Tobacco (SRNT) workgroup [42] for measuring smoking cessation. Seven-day smoking cessation will be defined as abstinent for the past seven days at one, six and 12 months respectively. Seven-day point prevalence abstinence is a common and recommended cessation outcome and has the highest concurrent validity of a range of cessation measures [43]. ***Biochemical *v*erification of self-report: ***General population reports and some clinical trial based studies show that misreporting tends to be low, around 5% [38]. Little is known regarding the misreporting rate of smoking status in this population. Thus, this study will use biochemical validation methods to ensure self-report estimates are validated. Verification will be conducted using measures of CO in expired air as recommended by the SRNT Biochemical Verification workgroup [44]. Although cotinine measures provide the gold standard tool for assessing smoking status self-report, the collection of blood, urine or saliva samples with clients in a non-health setting is invasive and impractical. To assess 24-hour smoking cessation, clients will be asked how long ago they last smoked a cigarette, cigar or pipe? (and respond by indicating, last four hours, between 4-8 hours ago, between 8-24 hours ago, more than 24 hours ago). Twenty four-hours is the recommended biochemically verifiable window for CO in expired air [44]. The Jarvis protocol will be used to record expired air CO. A cut-off point of 8 ppm will be used to define a smoker [44]. Measuring CO in expired air has demonstrated high sensitivity and specificity of around 90% for both [44].

#### Secondary outcome measure

In addition to the point prevalence measures, six and 12 month continuous abstinence will be assessed at six and 12 month follow up as recommended by the SRNT [42].

#### Moderating factors

These items will be collected at baseline, used as part of the intervention print-out, and included in multivariate analysis. ***Sociodemographic characteristics ***will include age, gender, marital status, housing status, income, education, postcode. ***Nicotine dependence ***will be measured by the Heaviness of Smoking Index (HSI), a two-item short form of the Fagerstrom Tolerance Questionnaire [45]. The HSI scores range from 0-6 and are calculated by summing the points for 1) time to first cigarette smoked after waking (in minutes) and 2) number of cigarettes smoked per day. Higher HSI scores indicate more dependence on nicotine. ***Quit attempts***. A commonly used [16,17] item which asks "How many serious attempts to stop smoking have you made in the last 12 months? By serious attempt I mean you decided that you would try to make sure you never smoked again. Please include any attempt that you are currently making and please include any successful attempt made within the last year". ***Use of cessation aids***. Clients who have made a serious quit attempt will be asked about treatments they had used in their most recent quit attempt. A single item developed by Shiffman et al [16] will list various pharmacological, behavioural and alternative smoking cessation aids. ***Partner smoking behaviour***. One item [46] will assess partner smoking behaviour: "Does your partner smoke?" (response options: yes, yes but stopping with me, no ex-smoker, no never smoked, N/A). ***Depression***. A recent meta-analysis indicates that the Patient Health Questionnaire (PHQ) has sensitivity of 80% and specificity of 92% in diagnosing major depression [47]. The two-item PHQ2 is recommended by the US Preventive Services Taskforce for depression screening and will ask "Over the last 2 weeks have you felt down, depressed or hopeless?" and ".. have you felt little interest or pleasure in doing things?". ***Financial stress ***will be measured using a scale developed by Siahpush et al [9,48] and previously used as a predictor of smoking behaviour. It includes items asking "In the last six months, did any of the following happen to you because of a shortage of money?" followed by six options.

#### Process measures. *Acceptability of intervention*

At six-month follow-up staff and clients will also be asked to rate the acceptability, usefulness and perceived effectiveness of the smoking cessation intervention. ***Staff and client intervention checklists. ***Completed staff and client printed checklists of intervention strategies offered, accepted, refused, and provided will be compared at the conclusion of the trial to measure exposure and adherence to the intervention. ***Costs. ***Relevant costs of delivery of the intervention and estimates of costs to the community service sector including staff time will be identified, collected and valued to allow a cost-benefit analysis from the service provider perspective. Savings in terms of cessation rates and savings in health care costs will be analysed.

### Data Analysis

Data will be stored in Access databases and exported for analysis to SAS. The distribution of all variables will be checked for normality, with non-parametric statistics used when appropriate. Characteristics of study participants will be compared between intervention groups using the chi-square test for categorical variables and the t-test or a non-parametric equivalent for continuous variables.

#### Efficacy of intervention

To assess the efficacy of the intervention at smoking cessation (validated by expired air CO), the intervention and control groups will be compared on each of the primary outcomes using chi-square analysis: 1) proportion abstinent last 24 hours (validated) at one, six and 12 months; and 2) proportion abstinent for seven days at one, six and 12 months. Univariate analyses will be undertaken to examine factors associated with each of the two outcomes, using the chi-square test for categorical variables and the t-test or a non-parametric equivalent for continuous variables. In addition to the primary analysis, intention-to-treat analysis will also be undertaken whereby clients who fail to complete follow-up in the study will be considered to be continuing smokers. Multiple logistic regression analyses will be used to compare outcomes between the two intervention groups while adjusting for prognostic variables and potential confounders (sociodemographic, financial stress, depression, and other variables collected).

#### Acceptability

The proportion of participants reporting that the intervention was acceptable, useful, and effective will be determined with 95% confidence intervals.

## Discussion

Notwithstanding the challenges of research with hard-to-reach populations, well-designed randomised controlled trials are essential for testing hypotheses with strong theoretical underpinnings to produce high quality evidence. This study is significant in a number of ways. It targets a particularly vulnerable group in the population that has been resistant to anti-tobacco campaigns and strategies to date. The reduction of the social inequity in smoking rates is increasingly recognised as a priority by peak cancer and health organisations. Secondly, it will be the first methodologically stringent study to investigate the efficacy of a smoking care intervention with disadvantaged groups in Australia. Previous research in this area is marked by poor methodological design. Thirdly, the intensive, client-focussed approach to the intervention has the potential to produce an effect size higher than those reported in the international literature to date. In designing the intervention, the various interacting influences on smoking amongst disadvantaged groups were acknowledged. Finally, a setting readily accessible and acceptable to disadvantaged groups is being tested. If effective, the intervention has clear potential for implementation in standard care in community social services. This study will generate new knowledge in an area where the current information regarding the most effective cessation approach with disadvantaged groups is limited.

## Appendix 1

### Description of the content of the case-worker smoking cessation intervention sessions

#### Case-worker session 1

At the clients first visit during an intervention week, staff will receive the *survey print-out and checklist *alerting them to client smoking status, nicotine dependence, whether and how they have attempted to quit in the past and other issues such as depression or financial stress which may require additional support.

##### Encouraging a quit attempt

All clients will be advised to quit. West et al [49] have found that spontaneous quit attempts that involve a decision to cease immediately can be just as successful than those where planning and preparation have taken place. Staff are familiar with techniques such as *motivational interviewing *[40] to assist their clients in coming to positive decisions. At that initial visit, clients will be asked to sign a *behavioural *contract [50] outlining the support they will be given by staff to help quit smoking and their role in compliance with use of support strategies and making quit attempts.

##### Use of effective cessation support strategies

A major component of the intervention is the *provision of pharmacotherapy. *Evidence suggests that smokers from lower socioeconomic backgrounds have higher levels of nicotine dependence [3] and that pharmacotherapy is an essential component of cessation care [11]. Clients will be offered a choice of medication at no cost. Nicotine replacement therapy almost doubles the chance of quit success compared with placebo [18]. A simple, safe and user-friendly version of the Bittoun [25,26] algorithm for appropriate use of multiple NRTs will be offered to clients with high nicotine dependence. Local pharmacists will also be provided with the algorithm and suggested as a source of advice and monitoring. There is evidence that the ability to quit smoking and maintain cessation is influenced by social support [18,46]. The case worker will ask the client to nominate a *'support' person *for their quit journey and provide a *support pack *to give to their support person. The support pack will contain advice on support strategies (e.g. advice on supportive behaviour, committing to an attempt to quit together until successful, not smoking near them, an NRT discount voucher if a smoker, and the Quitline number). If a client does not have a potential support person, the caseworker will locate a volunteer at the Centre who will take on the role of the support person via telephone contact.

##### Support for other potential relapse-related factors

As relevant, clients will be provided with information about courses and support options offered by the centre and other local agencies for issues such as depression and financial stress.

#### Case-worker sessions 2

At the next visit, smokers who have attempted to quit will be asked about their progress.

##### Encouraging a quit attempt

Motivational interviewing will be used to encourage additional quit attempts among those who have tried and failed; and to encourage either an immediate or pre-planned quit attempt prior to the next session (within the next two weeks).

##### Use of Effective Cessation Support Strategies

Continued use of NRT will be encouraged. Clients will be advised about other medications available on prescription to maximise their chances of successful quitting including bupropion SR, and varenicline. They will also be provided with written materials to take to their doctor. Those who need further assistance such as *referral to specialised services *will be offered a fax or email referral to Quitline, whereby the Quitline will call them.

##### Support for other potentially relapse-related factors

Clients experiencing stressful situations (mental health, financial or relationship concerns), as recorded at the survey print-out will be offered enrolment into *Life Skills courses *provided by the Centre. Other support options in the local area will be discussed. There is evidence that individuals from lower socioeconomic positions tend to have greater needs for a variety of life stressors including housing needs, financial stress, employment concerns and physical and mental health concerns which affect relapse [9] They will also be offered the opportunity to schedule a visit together with their support person. At the final scheduled face to face meeting, clients will be asked to drop-in or phone-in when they require assistance. They will be told that their counsellor will make further phone contact to monitor progress and address relapse.

#### Phone contacts (× 2)

*Maintenance and follow-up *are important components of this intervention. When face to face visits finish, staff will phone clients and check on progress, address difficulties, identify needs and provide advice. If practical aids are required, the client will be asked to return to the clinic to collect them. The invitation of *un-scheduled drop-in or phone-in sessions *will be reinforced. Springett and Owens [51] note that a client-led approach which included a drop-in system where clients were not required to pre-book appointments was valued and acceptable to disadvantaged clients of a stop smoking service and increased retention to the program and in some cases quit rates.

## Competing interests

The authors declare that they have no competing interests.

## Authors' contributions

BB led the writing of the original proposal and this manuscript. All authors contributed to the design of the study. BB, CP, RW contributed to the development of the intervention. BB, CD, and RC designed the statistical and economic analyses. BB, CP, RW and MS developed the outcome measures. All authors read and approved the manuscript.

## Pre-publication history

The pre-publication history for this paper can be accessed here:

http://www.biomedcentral.com/1471-2458/11/70/prepub
